# Draft Genome Sequence of *Pectobacterium wasabiae* Strain CFIA1002

**DOI:** 10.1128/genomeA.00214-14

**Published:** 2014-05-15

**Authors:** Kat (Xiaoli) Yuan, Zaky Adam, James Tambong, C. André Lévesque, Wen Chen, Christopher T. Lewis, Solke H. De Boer, Xiang (Sean) Li

**Affiliations:** a Canadian Food Inspection Agency, Charlottetown Laboratory, Charlottetown, PE, Canada; b Agriculture and Agri-Food Canada, Ottawa, ON, Canada

## Abstract

*Pectobacterium wasabiae*, originally causing soft rot disease in horseradish in Japan, was recently found to cause blackleg-like symptoms on potato in the United States, Canada, and Europe. A draft genome sequence of a Canadian potato isolate of *P. wasabiae* CFIA1002 will enhance the characterization of its pathogenicity and host specificity features.

## GENOME ANNOUNCEMENT

*Pectobacterium* spp. cause soft rot disease of a wide range of crops and ornamental plants both in the field and in storage, resulting in significant global economic losses in agricultural production (1). Species of the genus *Pectobacterium* were previously classified as *Erwinia*, a genus containing numerous species and subspecies of phytopathogenic bacteria varying in molecular and biochemical characteristics and host range. *Pectobacterium* spp. are characterized by their ability to produce large quantities of pectolytic enzymes involved in the maceration of parenchymal tissue of their plant hosts (2).

*Pectobacterium wasabiae* (formerly *Erwinia carotovora* subsp. *wasabiae*) was originally described as causing soft rot of Japanese horseradish (3) and was later identified as the causal agent of potato tuber decay in New Zealand (4, 5), the United States (6), and Iran (7). A recent study demonstrated that *P. wasabiae* also causes blackleg-like symptoms in potato plants (8). The pathogen possesses diverse regulatory systems with known virulence factors, including genes encoding pectolytic enzymes and the type III secretion system (T3SS), and it has many additional pathogenicity and virulence determinants acquired by horizontal gene transfer (9, 10). Therefore, comparative genomics of *P. wasabiae* strains infecting potato and other hosts from different geographical locations would help identify the specific virulence factors involved in pathogenicity and host specificity.

*P. wasabiae* strain CFIA1002 was isolated from a blackleg-diseased potato stem sample in Canada (8). The draft genome sequences of *P. wasabiae* strains WPP163 (11) and SCC3193 (1, 12), isolated from infected potato tubers in the United States and Europe, respectively, and the type strain *P. wasabiae* CFBP3394, isolated from horseradish in Japan, are available at GenBank (13, 14). The draft genome sequence data for *P. wasabiae* strain CFIA1002 were generated using paired-end Illumina HiSeq sequencing technology with TruSeq version 3 chemistry at the National Research Council Canada (Saskatoon, Saskatchewan, Canada). Sequencing resulted in 8,682,640 reads (insert size, 300 bp) totaling 876,946,640 bp, each 101 bp in length. The sequencing data provided approximately 175× genome coverage. After quality checking using FastQC (http://www.bioinformatics.babraham.ac.uk/projects/fastqc/), initial *de novo* assembly using ABySS (15) produced 78 contigs contained in 69 scaffolds, of which scaffolds with lengths of <300 bp were removed. SSPACE (16) and GapFiller (17) were applied on the scaffolds to extend and merge them into larger scaffolds and to close the gaps between the short scaffolds. The final draft genome is 5,008,535 bp in length, with 324 Ns, and consists of 42 scaffolds. The G+C content of the draft genome is 50.59%.

Annotation conducted on the RAST server using the Glimmer 3 option (18) predicted 4,615 protein-coding genes (96 noncoding RNAs). A number of predicted virulence factors, phage loci, and motility and chemotaxis genes were identified, which may facilitate pathogenicity in specific environments. The variable genomic regions, especially pathogenicity-related loci, were highly correlated with different environmental factors, including the host species. Further comparison of the genome sequences of strains from different hosts and geographic regions will provide further insights on virulence, functionality, and plant/pest interactions, as well as contribute to the development of specific assays for accurate identification and detection of the pathogen.

### Nucleotide sequence accession number.

This draft genome sequence of *P. wasabiae* strain CFIA1002 has been deposited in the DDBJ/EMBL/GenBank database under the accession no. JENG00000000. The version described in this paper is the ﬁrst version.
